# Incidence and Risk Factors of Postoperative Urinary Retention After Thyroidectomy: A Retrospective Cohort Study

**DOI:** 10.1002/wjs.70291

**Published:** 2026-03-04

**Authors:** Jee In Chang, Man Hon Tang, Moon Young Oh, Mira Han, Jung Man Lee, Hyeong Won Yu, Su‐Jin Kim, Young Jun Chai, June Young Choi

**Affiliations:** ^1^ Department of Surgery Seoul National University College of Medicine Seoul Korea; ^2^ Department of Surgery Woodlands Health Singapore Singapore; ^3^ Department of Surgery Seoul National University College of Medicine Seoul Metropolitan Government—Seoul National University Boramae Medical Center Seoul Korea; ^4^ Medical Research Collaborating Center Seoul Metropolitan Government Seoul National University Boramae Medical Center Seoul Korea; ^5^ Department of Anesthesia and Pain Medicine Seoul National University College of Medicine Seoul Metropolitan Government ‐ Seoul National University Boramae Medical Center Seoul Korea; ^6^ Department of Surgery Seoul National University Bundang Hospital Seongnam‐si Korea; ^7^ Transdisciplinary Department of Medicine & Advanced Technology Seoul National University Hospital Seoul Korea

**Keywords:** risk factors, thyroidectomy, urinary retention

## Abstract

**Background:**

Postoperative urinary retention (POUR) is a common complication, but its incidence and risk factors after thyroidectomy are not well‐defined. We investigated the incidence of POUR after thyroidectomy and its risk factors.

**Methods:**

We conducted a retrospective review of 511 consecutive patients who underwent thyroidectomy by a single surgeon. POUR was defined as the inability to void within 6 h of the last preoperative void performed immediately prior to transfer to the operating room, with bladder volume > 500 mL requiring catheterization. Univariable and multivariable logistic regressions were performed separately for male and female.

**Results:**

Among 511 patients (368 females, 143 males; mean age 49.6 years), 412 (80.6%) underwent lobectomy, 71 (13.9%) total thyroidectomy without lateral neck dissection, and 28 (5.5%) total thyroidectomy with lateral neck dissection. Surgical access was open in 333 (65.2%), transoral robotic in 158 (30.9%), and transoral endoscopic in 20 (3.9%). Overall, 68 patients (13.3%) developed POUR. In males, independent predictors were benign prostatic hyperplasia (BPH) (adjusted odds ratio [aOR] 7.890; 95% confidence interval [CI], 1.814–34.318; *p* = 0.006) and body mass index (BMI) < 25 kg/m^2^ (≥ 25 kg/m^2^ aOR 0.245; 95% CI, 0.066–0.909; *p* = 0.036; reference < 25 kg/m^2^), BMI < 25 kg/m^2^ (≥ 25 kg/m^2^ aOR 0.465; 95% CI, 0.228–0.947; *p* = 0.035; reference < 25 kg/m^2^), and operative time ≥ 60 min (aOR 1.939; 95% CI, 1.014–3.709; *p* = 0.045). Surgical approach, extent of surgery, pathology, and postoperative opioid use were not independently associated with POUR in either sex.

**Conclusions:**

POUR occurred in 13.3% of thyroidectomy patients. Sex‐stratified analysis showed BPH and lower BMI as key risks in males, whereas older age, lower BMI, and longer operative time were significant in females. Recognizing these factors may support targeted perioperative screening and postoperative monitoring to reduce retention‐related delays and complications.

## Graphical abstract

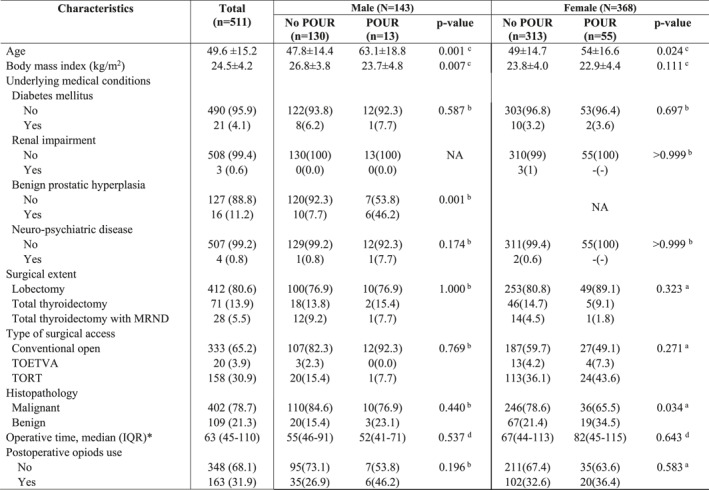

## 1 Introduction

Postoperative urinary retention (POUR) is a relatively common condition that can occur after surgery and anesthesia [1]. It is defined as the inability to urinate voluntarily despite a full bladder [2]. Although usually temporary, POUR can cause significant discomfort to patients and may lead to secondary complications such as urinary tract infection, bladder overdistension, and a prolonged hospital stay [3]. Therefore, even though it is considered a minor postoperative issue, proper recognition and management are clinically important.

The reported incidence of POUR varies widely among studies, depending on the type of surgery, anesthesia, and definition used [2, 4]. Several patient‐related factors have been associated with a higher risk of POUR, including older age, male sex, and the presence of benign prostatic hyperplasia (BPH), diabetes mellitus, or other lower urinary tract symptoms [5]. Operative and anesthetic factors such as surgical duration, postoperative pain, and the use of opioids are also known to contribute by altering the normal physiology of voiding through neural or pharmacologic mechanisms [6, 7].

Despite many studies addressing POUR in other surgical fields, there is limited information on its incidence and risk factors following head and neck surgery [8, 9]. However, to our knowledge, there are no published studies focusing specifically on POUR after thyroidectomy, which is the most common head and neck operation and is often performed as a short, low‐risk procedure or even as day surgery. Understanding the occurrence and associated factors of POUR in this setting is important for improving patient safety and optimizing postoperative care. Because male and female patients differ in anatomical and physiological aspects of the lower urinary tract, as well as in underlying risk factors such as benign prostatic hyperplasia and hormonal influences, the present study analyzed POUR risk factors separately for each sex to provide more accurate and clinically meaningful interpretations.

The present study aimed to investigate the incidence of postoperative urinary retention after thyroidectomy and to identify clinical and operative factors that may increase the risk of its development.

## 2 Material and Methods

### 2.1 Patients

This retrospective study was conducted at Seoul Metropolitan Government‐Seoul National University Boramae Medical Center and was approved by the Institutional Review Board (IRB No. 40‐2025‐60). Informed consent was waived due to the retrospective nature of the study. All consecutive patients who underwent thyroidectomy performed by a single endocrine surgeon (Y.J.C.) between July 2023 and June 2025 were included. Surgical procedures consisted of conventional open thyroidectomy and transoral remote‐access approaches, including both endoscopic and robotic techniques. Patients who underwent lobectomy, total thyroidectomy, and central or lateral neck dissection were all eligible for inclusion, regardless of surgical indication or approach. Patients with end‐stage renal disease who do not produce urine were excluded.

### 2.2 Surgical Procedure and POUR Protocol

All patients were admitted the day before surgery and fasted from midnight. During the fasting period and intraoperatively, intravenous Hartmann's solution was administered at a standardized maintenance rate of 80 mL/hr, irrespective of individual patient body weight. As part of the institutional preoperative protocol, patients were instructed to void immediately prior to transfer to the operating room, and the time of this last preoperative void was used as the reference point for postoperative urinary retention monitoring.

Following surgery, patients were allowed oral intake and mobilized as tolerated. Pain control consisted of regular oral paracetamol and as‐needed tramadol, unless contraindicated. POUR was monitored by nursing staff. If a patient was unable to void within 6 h of the last preoperative void or reported suprapubic discomfort, a portable bladder scan (CUBEscan BioCon‐500, Mcube Technology Co. Ltd., Seoul, Korea) was performed. Catheterization was performed if the bladder volume exceeded 500 mL. Urinary catheters were typically removed the next morning.

### 2.3 Anesthesia Protocol

All thyroidectomy procedures were performed under general anesthesia. The patients were admitted to the operating theater without any premedication on the day of surgery. Standard monitoring including peripheral oxygen saturation, noninvasive blood pressure, electrical cardiography, and bispectral (BIS) monitoring started. Induction of anesthesia was performed by the initiation of intravenous continuous infusion of propofol and remifentanil with target‐controlled infusion system after the administration of 1% lidocaine of 30 mg. After loss of consciousness, rocuronium of 0.6 mg/kg was administered for muscle relaxation to obtain the condition for tracheal intubation while the patient's lungs were manually being ventilated. Tracheal intubation was performed by using a video‐laryngoscope and a stylet with the patient's neck extended by a standard pillow on the patient's back to place the tube and maintain its position optimally for intraoperative neuromonitoring [10]. Next, the patient's lungs were ventilated mechanically with a tidal volume of 8 mL/kg and a rate of 8–12 to maintain the end‐tidal carbon dioxide to approximately 35–40 mmHg. The effect‐site concentration of propofol and remifentanil was adjusted according to the BIS level and hemodynamics such as blood pressure and heart rate, respectively, during the surgery. Rocuronium was not administered after the induction of anesthesia because of intraoperative neuromonitoring for all patients. Intraoperative crystalloid administration varied by patient but routinely ranged from 400 to 1200 mL. No patients required intraoperative Foley catheter placement.

For the open surgery, the combination of neostigmine of 2 mg and glycopyrrolate of 0.4 mg was administered at the beginning of the surgery for intraoperative neuromonitoring [11]. For the endoscopic surgery, the combination of neostigmine of 2 mg and glycopyrrolate 0.4 mg was administered at the end of surgery for emergence from anesthesia because the time from the administration of rocuronium at the induction of anesthesia to initiation of neuromonitoring during surgery was longer in the endoscopic surgery than the open surgery, which was efficient to recover muscle tone for intraoperative neuromonitoring. At the end of surgery, ramosetron 0.3 mg was administered to prevent postoperative nausea and vomiting for all patients.

### 2.4 Outcome Measures

Patient demographics, medical history, type of surgery, surgical access, operative time, and analgesic use were extracted from electronic medical records. POUR was defined, according to our institutional protocol, as the inability to void within 6 h of the last preoperative void with a bladder volume > 500 mL on portable bladder ultrasonography requiring catheterization.

### 2.5 Statistical Analyses

Continuous variables were summarized as mean ± standard deviation (SD) or median (Q1–Q3), and categorical variables were presented as frequencies and percentages. The normality of continuous variables was assessed using the Shapiro–Wilk test and further evaluated by visual inspection of Q–Q plots and histograms. Depending on the results of normality testing, comparisons were conducted using either the parametric *t*‐test or the nonparametric Wilcoxon rank‐sum test. Categorical variables were compared using the chi‐squared test, and Fisher's exact test was applied when more than 20% of the expected cell counts were less than 5.

Univariable and multivariable logistic regressions analyzed were performed to identify risk factors for POUR. The results of the analysis are presented as odds ratios (ORs) with 95% confidence intervals (95% CI). The Hosmer and Lemeshow Goodness‐of‐Fit Test was applied to assess the model's fit. In a multivariable analysis of the entire population, all variables except BPH were initially included, and backward elimination was applied to retain variables with *p* < 0.05 and clinically important variables such as age and operation time. Since BPH is a risk factor observed only in male patients, sex‐stratified logistic regression analyses were performed. All statistical analyses were performed using SAS software, version 9.4 (SAS Institute Inc., Cary, NC, USA). A two‐tailed *p*‐value of < 0.05 was considered statistically significant.

## 3 Results

A total of 511 patients who underwent thyroidectomy were included in the final analysis, comprising 368 females and 143 males. The mean age was 49.6 ± 15.2 years, and the mean body mass index (BMI) was 24.5 ± 4.2 kg/m^2^. A total of 412 (80.6%) patients underwent lobectomy, 71 (13.9%) patients underwent total thyroidectomy without lateral neck dissection, and 28 (5.5%) patients underwent total thyroidectomy combined with lateral neck dissection. In terms of surgical access, 333 (65.2%) procedures were performed via the conventional open approach, 158 (30.9%) through the transoral robotic approach, and 20 (3.9%) using the transoral endoscopic vestibular approach. Malignancy accounted for 402 (78.7%) cases of final pathology, whereas 109 (21.3%) cases were benign lesions. The median operative time was 63 min (interquartile range, 45–110), and postoperative opioids were administered in 163 patients (31.9%). POUR occurred in 68 patients, representing an overall incidence of 13.3%. Sex‐stratified comparisons of patient and operative characteristics by POUR status are shown in Table 1.

**TABLE 1:** Patient characteristics.

Characteristics	Total (*n* = 511)	Male (*N* = 143)			Female (*N* = 368)		
		No POUR (*n* = 130)	POUR (*n* = 13)	*p*‐value	No POUR (*n* = 313)	POUR (*n* = 55)	*p*‐value
Age	49.6 ± 15.2	47.8 ± 14.4	63.1 ± 18.8	0.001^d^	49 ± 14.7	54 ± 16.6	0.024^d^
Body mass index (kg/m^2^)	24.5 ± 4.2	26.8 ± 3.8	23.7 ± 4.8	0.007^d^	23.8 ± 4.0	22.9 ± 4.4	0.111^d^
Underlying medical conditions							
Diabetes mellitus							
No	490 (95.9)	122 (93.8)	12 (92.3)	0.587^c^	303 (96.8)	53 (96.4)	0.697^c^
Yes	21 (4.1)	8 (6.2)	1 (7.7)		10 (3.2)	2 (3.6)	
Renal impairment							
No	508 (99.4)	130 (100)	13 (100)	NA	310 (99)	55 (100)	> 0.999^c^
Yes	3 (0.6)	0 (0.0)	0 (0.0)		3 (1)	‐(‐)	
Benign prostatic hyperplasia							
No	127 (88.8)	120 (92.3)	7 (53.8)	0.001^c^	NA		
Yes	16 (11.2)	10 (7.7)	6 (46.2)				
Neuro‐psychiatric disease							
No	507 (99.2)	129 (99.2)	12 (92.3)	0.174^c^	311 (99.4)	55 (100)	> 0.999^c^
Yes	4 (0.8)	1 (0.8)	1 (7.7)		2 (0.6)	‐(‐)	
Surgical extent							
Lobectomy	412 (80.6)	100 (76.9)	10 (76.9)	1.000^c^	253 (80.8)	49 (89.1)	0.323^b^
Total thyroidectomy	71 (13.9)	18 (13.8)	2 (15.4)		46 (14.7)	5 (9.1)	
Total thyroidectomy with MRND	28 (5.5)	12 (9.2)	1 (7.7)		14 (4.5)	1 (1.8)	
Type of surgical access							
Conventional open	333 (65.2)	107 (82.3)	12 (92.3)	0.769^c^	187 (59.7)	27 (49.1)	0.271^b^
TOETVA	20 (3.9)	3 (2.3)	0 (0.0)		13 (4.2)	4 (7.3)	
TORT	158 (30.9)	20 (15.4)	1 (7.7)		113 (36.1)	24 (43.6)	
Histopathology							
Malignant	402 (78.7)	110 (84.6)	10 (76.9)	0.440^c^	246 (78.6)	36 (65.5)	0.034^b^
Benign	109 (21.3)	20 (15.4)	3 (23.1)		67 (21.4)	19 (34.5)	
Operative time, median (IQR)^a^	63 (45–110)	55 (46–91)	52 (41–71)	0.537^e^	67 (44–113)	82 (45–115)	0.643^e^
Postoperative opioids use							
No	348 (68.1)	95 (73.1)	7 (53.8)	0.196^c^	211 (67.4)	35 (63.6)	0.583^b^
Yes	163 (31.9)	35 (26.9)	6 (46.2)		102 (32.6)	20 (36.4)	

*Note:* All values are presented as mean ± standard deviation or *n* (%).

Abbreviations: IQR, interquartile range; MRND, modified radical neck dissection; POUR, postoperative urinary retention; TOETVA, transoral endoscopic thyroidectomy vestibular approach; TORT, transoral robotic thyroidectomy.

^a^ Operative time across all surgical extent and surgical access.

^b^ Chi‐square test.

^c^ Fisher's exact test.

^d^ *T*‐test.

^e^ Wilcoxon rank sum test.

In the univariate analysis, among male patients, those who developed POUR were significantly more likely to have a history of BPH (adjusted odds ratio [aOR] 10.287; 95%, CI, 2.898–36.520, *p* < 0.001) and had lower BMI (≥ 25 kg/m^2^ aOR 0.235; 95% CI, 0.069–0.807, *p* = 0.021; reference < 25 kg/m^2^) compared to those without POUR. Age, surgical approach, extent of surgery, pathology, operative time, and opioid use did not differ significantly between the two groups. Among female patients, those with POUR were significantly older (aOR 1.807; 95% CI, 0.999–3.267, *p* = 0.050) and had lower BMI (≥ 25 kg/m^2^ aOR 0.475; 95% CI, 0.236–0.956, *p* = 0.037; reference < 25 kg/m^2^). POUR was also more common in females who underwent longer operations (median operative time 78 vs. 60 min, *p* = 0.041). There were no significant differences in surgical approach, extent of surgery, pathology, or opioid use (Tables 2, 3).

**TABLE 2:** Logistic regression model for POUR in male patients.

Variables		Male (*N* = 143)					
		Univariable			Multivariable		
		OR	95% CI	*p*‐value	OR	95% CI	*p*‐value
Age (ref: < 50)	≥ 50 age	3.167	(0.927, 10.816)	0.066	1.657	(0.384, 7.153)	0.499
BMI (ref: < 25)	≥ 25	0.235	(0.069, 0.807)	0.021	0.245	(0.066, 0.909)	0.036
Diabetes	Yes	1.271	(0.146, 11.040)	0.828			
Benign prostatic hyperplasia	Yes	10.287	(2.898, 36.520)	0.0003	7.890	(1.814, 34.318)	0.006
Surgical extent (ref: Lobectomy)	Total thyroidectomy	1.111	(0.225, 5.498)	0.897			
	Total thyroidectomy with MRND	0.833	(0.098, 7.090)	0.868			
Surgical type (ref: open)	TOETVA	< 0.001	(< 0.001, > 999.999)	0.988			
	TORT	0.446	(0.055, 3.623)	0.450			
Histology (ref: Benign)	Malignant	0.606	(0.153, 2.398)	0.476			
Operative time (ref: < 60)	≥ 60 min	1.133	(0.361, 3.557)	0.831	1.319	(0.368, 4.722)	0.671
Postoperative opioids use	Yes	2.327	(0.731, 7.401)	0.153			

Abbreviations: aOR, adjusted odds ratio; BMI, body mass index; CI, confidence interval; MRND, modified radical neck dissection; OR, odds ratio; TOETVA, transoral endoscopic thyroidectomy vestibular approach; TORT, transoral robotic thyroidectomy.

**TABLE 3:** Logistic regression model for POUR in female patients.

Variables		Female (*N* = 368)					
		Univariable			Multivariable		
		OR	95% CI	*p*‐value	OR	95% CI	*p*‐value
Age (ref: < 50)	≥ 50 age	1.807	(0.999, 3.267)	0.050	2.417	(1.273, 4.588)	0.007
BMI (ref: < 25)	≥ 25	0.475	(0.236, 0.956)	0.037	0.465	(0.228, 0.947)	0.035
Diabetes	Yes	1.143	(0.244, 5.365)	0.865			
Surgical extent (ref: Lobectomy)	Total thyroidectomy	0.561	(0.212, 1.484)	0.244			
	Total thyroidectomy with MRND	0.369	(0.047, 2.870)	0.341			
Surgical type (ref: open)	TOETVA	2.131	(0.648, 7.013)	0.213			
	TORT	1.471	(0.809, 2.673)	0.205			
Histology (ref: Benign)	Malignant	0.516	(0.278, 0.957)	0.036			
Operative time (ref: < 60)	≥ 60 min	1.513	(0.832, 2.754)	0.175	1.939	(1.014, 3.709)	0.045
Postoperative opioids use	Yes	1.182	(0.650, 2.150)	0.584			

Abbreviations: aOR, adjusted odds ratio; BMI, body mass index; CI, confidence interval; MRND, modified radical neck dissection; OR, odds ratio; TOETVA, transoral endoscopic thyroidectomy vestibular approach; TORT, transoral robotic thyroidectomy.

On multivariable analysis in males, BPH remained a strong independent risk factor for POUR (aOR 7.890; 95% CI, 1.814–34.318; *p* = 0.006), and BMI < 25 kg/m^2^ was also independently associated with POUR (aOR 0.245; 95% CI, 0.066–0.909; *p* = 0.036). Age ≥ 50 years and operative time ≥ 60 min were not significantly associated with POUR in this subgroup (Table 2). Multivariable analysis in females revealed that age ≥ 50 years (aOR 2.417; 95% CI, 1.273–4.588; *p* = 0.007), BMI < 25 kg/m^2^ (aOR 0.465; 95% CI, 0.228–0.947; *p* = 0.035), and operative time ≥ 60 min (aOR 1.939; 95% CI, 1.014–3.709; *p* = 0.045) were independently associated with POUR (Table 3).

## 4 Discussion

This study analyzed the incidence and sex‐specific risk factors of POUR in patients undergoing thyroidectomy. The overall incidence of POUR was 13.3%, which is comparable or slightly higher to that reported for other nonpelvic, surgical procedures under general anesthesia [9, 12]. Sex‐stratified multivariable analyses revealed that BMI < 25 kg/m^2^ was independently associated with POUR in both male and female patients. In males, BPH was the strongest independent risk factor, whereas in females, older age and longer operative time were also significantly associated with POUR. Although POUR was transient in most cases and resolved the next morning, our findings suggest that clinicians should be aware of these risk factors when evaluating postoperative recovery and discharge plans.

Low BMI was consistently associated with a higher risk of POUR across both male and female patients. Although POUR is often perceived as more common in patients with larger body habitus, several studies in general surgery and orthopedics have reported a higher incidence among patients with lower BMI [13, 14]. One possible explanation is that leaner patients may have less physiologic reserve and may be more sensitive to anesthetic drugs, particularly agents that suppress detrusor muscle activity. Although many anesthetic agents are dosed based on body weight, some medications such as muscle relaxants or sedatives are commonly administered in fixed amounts regardless of the patient's size. As a result, patients with lower BMI may receive a relatively greater dose per kilogram, potentially increasing the risk of prolonged detrusor inhibition and delayed recovery of the bladder function. This heightened sensitivity to anesthetic effects likely explains why BMI less than 25 kg/m^2^ emerged as an independent risk factor in both sexes [15].

Age was also found to be a significant risk factor, but only in female patients. Women aged 50 years or older had a higher incidence of POUR, which is in line with previous findings in spine and general surgery [5, 12, 14]. This may be due to age‐related physiological changes such as reduced detrusor contractility and impaired sensation of bladder fullness [16, 17] as well as hormonal changes, including the effect of menopause and estrogen deficiency on urethral tone and pelvic floor muscles [18]. Older patients are also more likely to experience delayed ambulation and altered pain perception, both of which can interfere with normal micturition [19].

In male patients, the presence of BPH was a strong independent predictor of POUR. This finding is consistent with earlier studies across different surgical fields, which have shown that BPH contributes to urinary retention, even in surgeries not directly involving the genitourinary tract [2, 5, 6, 9]. In our cohort, the odds of POUR in male patients with a known history of BPH were nearly eight times higher than those without. Interestingly, neither age ≥ 50 years nor operative time ≥ 60 min were significant predictors in the male subgroup, which may suggest that the impact of BPH dominates the risk profile for POUR in this population. Because thyroidectomy is sometimes performed in ambulatory or day‐surgery settings, and male patients with BPH may have an elevated risk of postoperative urinary retention, it may be reasonable to consider the preoperative assessment of lower urinary tract symptoms or closer postoperative monitoring in these patients [20, 21].

Operative time of 60 min or more was another significant factor associated with POUR, but only in female patients. Longer operations often lead to greater cumulative exposure to intravenous fluids and anesthetic medications, both of which can affect normal bladder function [22, 23]. Delayed ambulation after longer surgeries may further contribute to urinary retention [24]. In our data, operative time was correlated with malignant histology, suggesting that more extensive resections likely contribute to this risk. However, pathology itself did not remain significant in multivariable analysis, implying that it is the duration and intensity of surgery rather than the diagnosis that influences POUR development. These observations align with studies in head and neck as well as general surgery, where longer operative times have been consistently linked to increased POUR risk [6, 25].

Given the protocol‐driven nature of urinary retention surveillance, it is also important to interpret our incidence and associated risk factors in the context of the operational definition used at our institution. There is no universally accepted definition of POUR, and reported incidence varies widely across studies largely because diagnostic thresholds differ, including time‐to‐void criteria, bladder scan volume cutoffs, symptom‐based assessment, and catheterization requirements [26]. In particular, time‐based definitions commonly use a 6–8‐h window depending on the clinical setting and catheter practices, with some studies defining POUR as failure of spontaneous voiding 6 h after catheter removal, whereas others describe POUR as inability to void 8 h after the end of surgery [27, 28]. Our protocol defines POUR as the inability to void within 6 h of the last preoperative void, with bladder ultrasonography performed when indicated and catheterization triggered at a bladder volume exceeding 500 mL. This conservative approach may increase sensitivity compared with definitions that use higher volume thresholds, such as exceeding 600 mL combined with persistent inability to void, or protocols that use individualized maximum bladder capacity rather than a fixed cutoff [26]. Accordingly, the relatively higher POUR rate observed in our cohort is likely influenced by the early time checkpoint and the 500‐mL catheterization threshold, and this framework should be considered when comparing our results with other nonpelvic surgical populations.

This study has several limitations. First, it was a retrospective analysis based on a chart review. POUR data were obtained from nursing records, which may introduce variability. Specifically, the recorded time of voiding or catheterization, defined as 6 h from the last preoperative void immediately prior to transfer to the operating room, may not have been consistently or precisely documented. In addition, bladder residual volumes were measured using portable bladder scanners, which can vary depending on the nursing staff performing the scan. Second, POUR was defined according to the institutional protocol, which may differ from those used at other centers and may not capture transient or subclinical cases. Lastly, this study reflects the experience of a single high‐volume endocrine surgeon at a single institution, limiting the generalizability of the findings. Cultural and institutional differences in discharge practices and ambulation protocols may also influence the observed incidence of POUR.

In conclusion, POUR is a clinically relevant complication following thyroidectomy, with an incidence of over 13% in our cohort. Risk factors differed by sex, with BPH predominating in males, and older age, lower BMI, and longer operative time significantly associated with POUR in females. Although most cases resolved without long‐term sequelae, POUR can delay recovery and discharge, particularly in patients managed as same‐day cases. Identifying at‐risk individuals preoperatively and implementing targeted monitoring protocols may help improve postoperative care. Prospective studies are warranted to determine whether interventions such as early ambulation, selective catheterization, or pharmacologic prophylaxis may reduce the risk of POUR in thyroidectomy patients.

## Author Contributions

**Jee In Chang:** data curation, investigation. **Man Hon Tang:** conceptualization, data curation, writing – original draft. **Mira Han:** methodology, formal analysis. **Jung Man Lee:** methodology. **Su‐Jin Kim:** investigation, resources. **Young Jun Chai:** conceptualization, investigation, writing – review and editing.

## Funding

The authors have nothing to report.

## Ethics Statement

This retrospective study was conducted at Seoul Metropolitan Government‐Seoul National University Boramae Medical Center and was approved by the Institutional Review Board (IRB No. 40‐2025‐60). Informed consent was waived due to the retrospective nature of the study.

## Conflicts of Interest

The authors declare no conflicts of interest.

## Data Availability

The data that support the findings of this study are available from the corresponding author upon reasonable request.
